# The Effectiveness of Pharmacological and Non-Pharmacological Interventions for Improving Glycaemic Control in Adults with Severe Mental Illness: A Systematic Review and Meta-Analysis

**DOI:** 10.1371/journal.pone.0168549

**Published:** 2017-01-05

**Authors:** Johanna Taylor, Brendon Stubbs, Catherine Hewitt, Ramzi A. Ajjan, Sarah L. Alderson, Simon Gilbody, Richard I. G. Holt, Prakash Hosali, Tom Hughes, Tarron Kayalackakom, Ian Kellar, Helen Lewis, Neda Mahmoodi, Kirstine McDermid, Robert D. Smith, Judy M. Wright, Najma Siddiqi

**Affiliations:** 1 Department of Health Sciences, University of York, York, United Kingdom; 2 Health Service and Population Research Department, Institute of Psychiatry, Psychology and Neuroscience, King's College London, London, United Kingdom; 3 Physiotherapy Department, South London and Maudsley NHS Foundation Trust, London, United Kingdom; 4 Leeds Institute of Cardiovascular and Metabolic Medicine, University of Leeds, Leeds, United Kingdom; 5 Leeds Institute of Health Sciences, University of Leeds, Leeds, United Kingdom; 6 Human Development and Health Academic Unit, Faculty of Medicine, University of Southampton, Southampton, United Kingdom; 7 Leeds and York Partnership NHS Foundation Trust, Leeds, United Kingdom; 8 School of Psychology, University of Leeds, Leeds, United Kingdom; 9 Faculty of Health and Social Sciences, Leeds Beckett University, Leeds, United Kingdom; 10 University of Leeds Library, Leeds, United Kingdom; 11 Bradford District Care NHS Foundation Trust, Bradford, United Kingdom; Providence VA Medical Center, UNITED STATES

## Abstract

People with severe mental illness (SMI) have reduced life expectancy compared with the general population, which can be explained partly by their increased risk of diabetes. We conducted a meta-analysis to determine the clinical effectiveness of pharmacological and non-pharmacological interventions for improving glycaemic control in people with SMI (PROSPERO registration: CRD42015015558). A systematic literature search was performed on 30/10/2015 to identify randomised controlled trials (RCTs) in adults with SMI, with or without a diagnosis of diabetes that measured fasting blood glucose or glycated haemoglobin (HbA_1c_). Screening and data extraction were carried out independently by two reviewers. We used random effects meta-analysis to estimate effectiveness, and subgroup analysis and univariate meta-regression to explore heterogeneity. The Cochrane Collaboration’s tool was used to assess risk of bias. We found 54 eligible RCTs in 4,392 adults (40 pharmacological, 13 behavioural, one mixed intervention). Data for meta-analysis were available from 48 RCTs (n = 4052). Both pharmacological (mean difference (MD), -0.11mmol/L; 95% confidence interval (CI), [-0.19, -0.02], p = 0.02, n = 2536) and behavioural interventions (MD, -0.28mmol//L; 95% CI, [-0.43, -0.12], p<0.001, n = 956) were effective in lowering fasting glucose, but not HbA_1c_ (pharmacological MD, -0.03%; 95% CI, [-0.12, 0.06], p = 0.52, n = 1515; behavioural MD, 0.18%; 95% CI, [-0.07, 0.42], p = 0.16, n = 140) compared with usual care or placebo. In subgroup analysis of pharmacological interventions, metformin and antipsychotic switching strategies improved HbA_1c_. Behavioural interventions of longer duration and those including repeated physical activity had greater effects on fasting glucose than those without these characteristics. Baseline levels of fasting glucose explained some of the heterogeneity in behavioural interventions but not in pharmacological interventions. Although the strength of the evidence is limited by inadequate trial design and reporting and significant heterogeneity, there is some evidence that behavioural interventions, antipsychotic switching, and metformin can lead to clinically important improvements in glycaemic measurements in adults with SMI.

## Introduction

People with severe mental illness (SMI) (schizophrenia and other illnesses characterised by psychosis) have a lower life expectancy compared with the general population by around 15 to 20 years [1]. A higher prevalence of comorbid conditions (e.g. diabetes and cardiovascular disease) and poorer management of physical health contribute to this health inequality [2]. Around 13% of people with SMI have diabetes compared with 6% of the general population, and the difference is increasing [3]. As diabetes interventions are scaled up for the general population, these inequalities may increase further. This is because generic interventions are unlikely to be suitable for people with SMI due to the complex combination of psychological, social and financial barriers they face in managing their health [4].

Although there are more than 40 published systematic reviews of studies targeting physical health in people with SMI, these have focused mainly on anthropological outcomes [5–8], with few investigating diabetes prevention and treatment [9, 10]. It is well-established that modest improvements in glycated haemoglobin (HbA_1c_) and blood glucose levels can avoid onset of diabetes and have a significant impact on preventing diabetic complications in the general population [11]. A few reviews have investigated the effect of pharmacological [6, 12] and behavioural [7, 8, 13] interventions on these glycaemic measurements in people with SMI. An older review investigated both pharmacological and behavioural interventions [14]. However in all of these, glycaemic effects were examined as a secondary outcome only. This makes it difficult to determine which interventions are effective for improving glycaemic control in people with SMI. The aim of this systematic review and meta-analysis is to identify pharmacological and behavioural interventions for improving diabetes outcomes that have been tested in the adult SMI population, and to determine their effectiveness in lowering HbA_1c_ and fasting blood glucose [15].

## Methods

### Eligibility criteria

We included randomised controlled trials (RCTs) of interventions to improve diabetes outcomes for adults (aged 18 years and over) with SMI. We defined SMI as schizophrenia, bipolar disorder, psychosis or other non-organic psychotic disorders, including schizoaffective disorder and severe depression. To be included, studies had to measure at least one of the following outcomes: i) in people without diabetes at baseline: incidence of diabetes, HbA_1c_ or fasting glucose; and ii) in people with diabetes at baseline: HbA_1c_, fasting glucose, weight, body mass index (BMI), or diabetic complications.

We restricted studies to those published in peer reviewed journals and the English language.

The protocol for the review has been published on the International Prospective Register of Systematic Reviews (PROSPERO), registration number CRD42015015558 [15]. We carried out the review in accordance with the PRISMA guidelines (see S1 PRISMA Checklist).

### Search strategy

The search strategy comprised three concepts: ‘diabetes’, ‘SMI’, and ‘RCTs or systematic reviews’. An example of the strategy is provided in the supporting information (see S1 Appendix).

Literature searches were performed in CINAHL (EBSCO); Embase Classic+Embase (Ovid); PsycINFO (Ovid); Ovid Medline; PubMed; Cochrane Database of Systematic Reviews (Wiley) and Central Register of Controlled Trials; Database of Abstracts of Reviews of Effect (Wiley); and Conference Proceedings Citation Index (Thomson Reuters). We also searched three trial registries (ClinicalTrials.gov, International Clinical Trials Registry Platform (WHO), ISRCTN registry). Searches were performed on 12/12/2014, and updated on 30/10/2015 (except for trial registries).

### Study selection

Search results were managed in EndNote version 7 software. Citations and abstracts were screened to exclude studies that did not meet the selection criteria. References of relevant reviews identified during the screening process were also searched. Relevant full-text articles were retrieved and assessed for eligibility; missing data to help assess eligibility were sought from corresponding authors.

### Data extraction and synthesis

Study characteristics and data for meta-analysis were extracted into a tailored and piloted data collection form [15]. Multiple reports from the same study were linked and missing data were requested from study authors. The Cochrane Collaboration tool was used to assess risk of bias [16]. All stages of study selection and data extraction were conducted independently by two reviewers, with discrepancies resolved through discussion and where consensus could not be reached, arbitration by a third reviewer.

Due to the heterogeneity of diabetes interventions, we categorised interventions as pharmacological, non-pharmacological or mixed (interventions combining medication with a non-pharmacological approach) [15]. Pharmacological interventions were further sub-grouped into categories: i) diabetes medications (including metformin, sulphonylureas, insulin and thiazolidinediones); ii) weight loss treatments (including antiparkinsonian, anticonvulsant and antidepressant medications thought to promote weight loss, as well as anti-obesity drugs and appetite suppressants); iii) combinations of weight loss and diabetes medications; iv) switching antipsychotic medication; and v) an ‘other’ category.

Non-pharmacological interventions were categorised as behavioural (targeting a change in an individual’s behaviour) or organisational (targeting a change in the environment or organisation of care).

We planned to explore effectiveness of interventions in prevention of diabetes. However, many studies did not distinguish between people with and without diabetes at baseline. Of the studies that excluded people with diabetes at baseline, none measured incidence of diabetes or reported data that would enable us to estimate this. We therefore pooled the results across all studies for glycaemic control, using outcome data for HbA_1c_ and fasting glucose.

We analysed pharmacological and non-pharmacological interventions separately, and because we expected significant heterogeneity between studies, we used random-effects meta-analysis and assessed for heterogeneity using the I-squared statistic. To allow combining of post-intervention and change scores for outcomes, and since outcomes were reported consistently, we calculated the unstandardised difference in means (MD) [16].

To assess effects across key intervention characteristics, we conducted subgroup analyses for pharmacological interventions by type of drug category; and for behavioural interventions by duration (short (≤6 months) or long (>6 months)), and whether or not interventions included repeated physical activity. We also conducted univariate random effects meta-regression using intervention duration as a continuous variable (number of weeks). Both duration and physical activity have been identified as key components of effective diabetes interventions in the general population [17].

To explore potential differential effects in people with and without diabetes, we conducted separate subgroup analyses, for i) studies excluding participants with diabetes, and ii) those that only included people with diabetes and SMI or did not specify diabetes status. We also conducted univariate random effects meta-regression using mean HbA_1c_ or fasting glucose at baseline to explore whether or not this explained some of the heterogeneity among studies [18].

To investigate possible baseline imbalance observed during data extraction, we repeated the main meta-analyses using mean difference at baseline [19]. We explored the impact of study quality and heterogeneity by undertaking sensitivity analyses, using ‘leave-one-out’ analyses to test if single studies had a disproportionate effect on the results. We used the trim-and-fill method and inspection of funnel plots to investigate publication and small study bias [20]. The trim-and-fill analysis adjusts for any funnel plot asymmetry and provides an effect size estimate that takes account of observed publication bias.

Comprehensive Meta-Analysis (CMA) version 2 software was used for all statistical analyses.

## Results

A total of 3,721 citations were identified by database searches, and a further 27 articles from the reference lists of systematic reviews. After removing duplicates, 2,278 records were screened for relevance by title and abstract, and 197 full text articles retrieved. Of these, 104 did not meet the selection criteria and were excluded. The remaining 93 articles described 73 studies. Nineteen of these were ongoing studies (see S1 Table).

**Fig 1:**
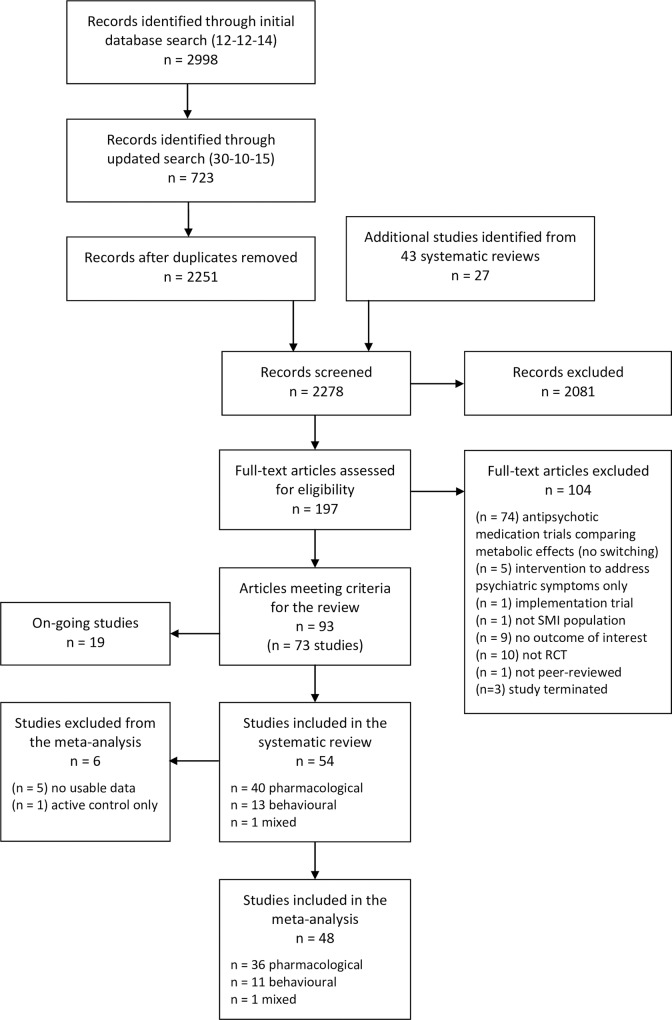
Study flow diagram.

A total of 54 studies were included in the systematic review [21–74]. Six of these studies did not provide usable data for the meta-analysis [26, 32, 42, 44, 46, 51]. Fig 1 presents a study flow diagram.

Study characteristics are summarised in Table 1.

**Table 1:** Characteristics of Included Studies.

Study ID and country	Study aim	Setting (number randomised)	SMI diagnoses	Diabetes diagnoses	Other relevant inclusion criteria	Intervention duration and frequency / dosage	Control	Follow-up time points^a^	Primary outcome^b^	Diabetes outcomes
**BEHAVIOURAL INTERVENTIONS**										
Attux 2013 [21], Brazil	Test efficacy of a lifestyle wellness intervention for weight gain management in schizophrenia	Patients attending four outpatient units (n = 160)	• Schizophrenia diagnosis • Using any APM for past 3 months • Clinically stable	Excluded patients with diabetes	• Age 18 to 65 • Excluded patients using weight loss medication • Motivation to lose weight	• Duration: 12 weeks • Manualised: YES • Group: 12 weekly 1-hour sessions • Leaders: trained MHP	Standard care	**3 months** • 6 months	**Change in body weight** ^**c**^	Fasting plasma glucose
Cordes 2014 [22], Germany	Investigate effects of weight loss programme on body weight and metabolic parameters in schizophrenia patients on OLZ	In-patients of one psychiatric facility (n = 74)	• Schizophrenia diagnosis • New treatment with OLZ • Weight gain of > 1.5kg in 4 weeks of taking OLZ	Excluded patients with diabetes	• Age 18 to 65 • Excluded weight gain of > 3kg in 3 months preceding • Excluded weight loss medication	• Duration: 24 weeks • Manualised: YES • Group: 12 bi-weekly 90-minute sessions • Leaders: Dietician with mental health counselling experience	Treatment as usual	**24 weeks** • 48 weeks	**Change in body weight** ^**d**^	**Fasting plasma glucose** ^**d**^**, OGTT** ^**d**^
Daumit 2013 [23], US	Determine effectiveness of behavioural weight-loss intervention in SMI	Patients of ten community outpatient psychiatric rehabilitation programmes (n = 291)	• Attending psychiatric rehabilitation programme • On same APM for 30 days prior to study		• Age 18 and older • BMI ≥ 25 • Excluded weight gain of > 20lbs in 3 months prior • Excluded weight loss medication	• Duration: 18 months • Manualised: YES • Group: 3 times per week for 6 months, then monthly sessions for 12 months • Individual: Yes if required • Leaders: trained study staff and rehabilitation staff	Nutrition and physical activity information at baseline, offered quarterly general health classes	• 6 months • 12 months • **18 months**	**Change in body weight**	Fasting blood glucose ^**e**^ (no p-value)
Forsberg 2008 [24], Sweden	Investigate effects of a lifestyle programme on metabolic syndrome and physiological parameters in psychiatric disability	Adults with a psychiatric disability receiving housing support or in supported housing (n = 46 ^**f**^**)**	• A psychiatric diagnosis in accordance with DSM-IV			• Duration: 12 months • Manualised: YES • Group: 2-hour study circle for 5–12 residents and staff held twice weekly for 12 months • Leaders: participating staff with no training	Controls offered an aesthetic study circle to learn artistic techniques, group-based weekly 2 hour sessions	• 12 months	Presence of MetS • **, mean no. criteria for MetS**	Fasting plasma glucose, HbA_1c_
Gillhoff 2010 [25], Switzerland	Evaluate effects of lifestyle intervention on BMI and cardiovascular and metabolic parameters in bipolar disorder	Outpatients of a psychiatric hospital and associated psychiatrists, study advertised in local press (n = 50)	• Bipolar disorder diagnosis • Receiving psycho-pharmacologic treatment for 3 months	Excluded patients with diabetes	• Age 18 to 70 • Not underweight (BMI > 20) • Excluded weight loss medication	• Duration: 5 monthsManualised: YES • Group: Weekly sessions for 11 weeks • Individual: Yes if required • Leaders: MHP, nutrition counsellor and fitness trainer	Standard care (wait list)	**3 months** • 11 months	**BMI**^**g**^	HbA_1c_
Goldberg 2013 [26], US **(excluded from meta-analysis)**	Evaluate effects of weight management programme on body weight in Veterans with SMI	Outpatients of Veteran Association mental health clinics (n = 109)	• DSM-IV diagnosis of schizophrenia, other psychotic spectrum disorder, bipolar disorder, major depression, or severe anxiety disorder	Excluded patients with elevated HbA_1c_ or fasting glucose levels	• Age 18 to 75 • BMI ≥ 25 • Excluded weight loss medication in 3 months prior to study	• Duration: 6 months • Manualised: YES • Group: Weekly sessions in months 2–4, biweekly in months 5–6 • Individual: Weekly sessions in month 1 • Leaders: Research staff	Offered basic information about diet and exercise each month	**3 months** • Plus interim monthly weigh-in	Weight loss	Fasting glucose
Green 2015 [27], US	Assess whether lifestyle intervention reduces weight and diabetes risk in individuals with SMI	Outpatients of community mental health centres and a not-for-profit integrated health plan (n = 200)	• Taking antipsychotic medication for ≥ 30 days prior to study		• Age 18 and older • BMI ≥ 27	• Duration: 6 months + 6 months maintenance • Manualised: YES • Group: Weekly 2-hour sessions in months 1–6, monthly in 7–12 • Individual: Monthly call / email in months 7–12 • Leaders: 2 facilitators (nutrition & counselling)	Usual care and free to initiate any weight loss effort on their own	• 6 months • **12 months** • 24 months	**Weight loss**	**Fasting plasma glucose**
Mauri 2008 [28], US	Evaluate efficacy of psycho-educational weight loss programme for patients with OLZ induced weight gain	Psychiatric outpatients (n = 45 ^**h**^**)**	• Receiving treatment of OLZ		• Age 18 to 65 • Increase of BMI (>7%) during treatment with OLZ	• Duration: 24 weeks • Manualised: Not reported • Sessions: Weekly 30-minute sessions for 24 weeks (not stated if group or individual) • Leaders: Not reported	Routine care in weeks 1–12, intervention in weeks 13–24	**12 weeks** • 24 weeks	**Weight loss**	Fasting plasma glucose
McKibben 2006 [29], US	Test feasibility and efficacy of lifestyle intervention for older patients with type 2 diabetes and schizophrenia	Residential care facilities, day programmes and community clubhouse settings (n = 64)	• Diagnosis of schizophrenia or schizoaffective disorder	Only included type 2 diabetes	• Age over 40 • Excluded patients with congestive heart failure	• Duration: 24 weeks • Manualised: YES • Group: Weekly 90-minute sessions for 24 weeks • Leaders: Diabetes-trained MHP	Usual care plus written information about managing diabetes	**3 months** • 12 months	**BMI**	Fasting plasma glucose, HbA_1c_
Poulin 2007 [30], Canada	Determine effectiveness of physical exercise programme for preventing APM-induced weight gain in patients with SMI	Outpatients of psychiatric departments in two hospitals (n = 130)	• DSM-IV diagnosis of schizophrenia, schizoaffective disorder or bipolar disorder • Current treatment with atypical APM		• Age 18 and over • Only included sedentary or moderately active patients	• Duration: 18 months • Manualised: NR • Group: One 90-minute counselling session followed by twice weekly 1-hour exercise sessions for 18 months • Leaders: Nutritionist, MH nurse, kinesiologist	Usual care	• 6 months • 12 months • **18 months**	**Change in body weight**	**Fasting plasma glucose, HbA**_**1c**_ ^**i**^
Scheewe 2013 [31], Netherlands	Examine effect of exercise vs OT on mental and physical health in schizophrenia patients	In-patients and out-patients of a University Medical Centre and three regional MH institutes (n = 63)	• DSM-IV diagnosis of schizophrenia, schizoaffective or schizophreniform disorder • Stable on APM		• No evidence of significant cardio-vascular disorder	• Duration: 6 months • Manualised: YES • Group: Twice weekly 1-hour sessions for 6 months • Leaders: Supervised by psychomotor therapist	• Active control: OT • Group: Twice weekly 1-hour sessions for 6 months • Individual: None • Leaders: OT	• 6 months	Mental health: Positive and negative symptoms **Physical health:CV fitness levels**	Fasting plasma glucose
Weber 2006 [32], US **(excluded from meta-analysis)**	Examine effectiveness of cognitive behavioural intervention for weight loss in schizophrenia patients taking APM	Outpatients attending a large urban public mental health clinic (n = 17)	• DSM-IV diagnosis of schizophrenia or schizoaffective disorder		• Age 18 to 65 • BMI ≥ 25	• Duration: 16 weeks • Manualised: YES • Group: Weekly 1-hour sessions for 16 weeks • Leaders: MH nurse practitioner	Treatment as usual	• Week 4 • Week 8 • Week 12 • **Week 16**	Weight loss	Fasting glucose ^j^
Wu 2007 [33], Taiwan	Evaluate effects of dietary control and physical activity for CLZ induced weight gain in schizophrenia patients	Inpatients of a veterans hospital (n = 53)	• DSM-IV diagnosis of schizophrenia • Receiving treatment of CLZ (>300mg daily for at least 1 year)		• Age 18 to 65 • BMI ≥ 27	• Duration: 6 months • Manualised: NR • Individual: Restricted caloric intake and physical activity of >30 minutes 3 times/week • Leaders: Registered dietician	NR	• 3 months • **6 months**	**Weight loss**	Serum fasting glucose
**MIXED INTERVENTIONS**										
Wu 2008 A [34] ^**k**^, China	Test efficacy of lifestyle intervention and / or metformin for APM induced weight gain in schizophrenia patients	Outpatient clinic of one regional hospital (n = 128), 4 arm trial	• First psychotic episode of schizophrenia (DSM-IV diagnosis) • Weight gain of > 10% within first year of APM treatment • Only taking CLZ, OLZ, risperidone, or sulpiride	Excluded patients with diabetes	• Age 18 to 45 • Under the care of parent or another adult caregiver	• Duration: 12 weeks • **A: Lifestyle PLUS placebo** • Manualised: NR • Group: Lifestyle sessions at baseline, weeks 4, 8, and 12 PLUS daily 30-minute exercise in week 1 • Leaders: Exercise physiologist and dietician • **B: Metformin** • Dose: 250mg/day for 4 days, then 750mg (250mg x 3)/day to week 12 • **C: Lifestyle AND metformin**	**D: Placebo**	• Week 4 • Week 8 • **Week 12**	**Change in body weight**	**Fasting glucose**
**PHARMACOLOGICAL INTERVENTIONS**										
Amrami-Weizman 2013 [35], Israel	Investigate effects of reboxetine on OLZ induced weight gain and metabolic parameters in schizophrenia patients	Inpatients of one mental health centre (pilot n = 26, trial n = 59 ^**l**^)	• First episode of schizophrenia (DSM-IV diagnosis) • Recommendation for OLZ treatment on hospitalization • < 4 weeks exposure to APM in preceding 6 months (no OLZ)	Excluded patients with diabetes	• BMI < 30 (i.e. excluded obese participants)	• Duration: 6 weeks • OLZ (10mg/day) PLUS Reboxetine • Dose: 2mg twice daily (4mg/day)	OLZ (10mg/day) PLUS Placebo	• Week 6	**Change in body weight**	Serum fasting glucose
Baptista 2006 [36], Venezuela	Assess whether metformin prevents body weight gain and metabolic dysfunction in patients with schizophrenia switched to OLZ	Inpatients of psychiatric rehabilitation unit (n = 40)	• DSM-IV diagnosis of schizophrenia or schizoaffective disorder • Stable for more than 5 years with conventional APM • Switching to OLZ	Free of any other chronic disease		• Duration: 14 weeks • OLZ (10mg/day) PLUS Metformin • Dose: 850 to 1750mg/day	OLZ (10mg/day) PLUS Placebo	• Week 7 • **Week 14**	Change in body weight	Fasting glucose, OGTT
Baptista 2007 [37], Venezuela	Assess whether metformin reverses OLZ induced weight gain and metabolic dysfunction in patients with SMI	Out- and inpatients in three states of Venezuela (n = 80)	• DSM-IV diagnosis of schizophrenia or bipolar disorder • Receiving treatment with OLZ (5–20mg daily for more than 4 months preceding)	Free of any other chronic disease	• Age 18 and over • Be willing to lose weight or prevent excessive body weight gain	• Duration: 12 weeks • Metformin • Dose: 850mg daily increased up to 2250mg by week 4 (adjusted according to individual tolerance)	Placebo	• Week 12	Weight loss	HbA_1c_, fasting serum glucose
Baptista 2008 [38], Venezuela	Assess whether metformin plus sibutramine reverses OLZ induced weight gain and metabolic dysfunction in patients with schizophrenia	Inpatients of psychiatric rehabilitation unit (n = 30)	• DSM-IV diagnosis of schizophrenia • Stable for more than 5 years • Switched from conventional APM to OLZ monotherapy 4 months prior to study	Free of any other chronic disease	• Age 18 and over • Be willing to lose weight or prevent excessive body weight gain	• Duration: 12 weeks • Metformin plus sibutramine • Dose: Week 1 to 4–850mg metformin plus 10mg sibutramine daily, week 5 to 12–850mg metformin plus 10mg sibutramine twice daily	Placebo	• Week 12	Change in body weight	HbA_1c_, fasting glucose
Baptista 2009 [39], Venezuela	Assess the effects of rosiglitazone on body weight and metabolic parameters in patients with schizophrenia receiving OLZ	Inpatients of psychiatric rehabilitation unit (n = 30)	• DSM-IV diagnosis of schizophrenia • Stable for more than 5 years • Switched from conventional APM to OLZ monotherapy 8 months prior to study	Free of any other chronic disease	• Age 18 and over • Be willing to lose weight or prevent excessive body weight gain	• Duration: 12 weeks • Rosiglitazone • Dose: 4mg daily increased up to 8mg daily from week 4	Placebo	• Week 6 • **Week 12**	Change in body weight	HbA_1c_, fasting serum glucose
Biedermann 2013 [40], Austria	Investigate effect of sibutramine for APM induced weight gain in patients with schizophrenia	Outpatients (n = 15)	• Diagnosis of schizophrenia (following ICD-10) • Receiving APM treatment		• Age 19 to 65 • BMI > 27 • Gained at least 7% body weight during APM treatment	• Duration: 24 weeks • Sibutramine • Dose: 10mg daily	Placebo	• Week 12 • **Week 24**	**Change in body weight**	**HbA**_**1c**_, random glucose
Borba 2011 [41], US	Examine the effects of ramelteon for APM induced weight gain and metabolic disturbances in patients with schizophrenia	Outpatients of one mental health centre (n = 25)	• DSM-IV diagnosis of schizophrenia or schizoaffective disorder • Clinically stable • Receiving APM treatment of CLZ, OLZ, risperidone or quetiapine	Excluded patients with diabetes or diabetic fasting glucose levels	• Age 18 to 65 • BMI > 27 • Evidence of insulin resistance or MetS • Excluded patients using weight loss medication	• Duration: 8 weeks • Ramelteon • Dose: 8mg daily	Placebo	• Week 8	Change in body weight	HbA_1c_, fasting plasma glucose
Borovicka 2002 [42], US **(excluded from meta-analysis)**	Investigate the effects of PPA for managing CLZ induced weight gain in patients with schizophrenia	Outpatients of a CLZ treatment programme (n = 16)	• DSM-IV diagnosis of schizophrenia • On stable dose of CLZ for > 4 months	Excluded patients with diabetes	• > 10% increase in body weight since starting CLZ • Excluded previous weight loss medication	• Duration: 12 weeks • PPA • Dose: 75mg sustained-release daily	Placebo	• Week 4 • Week 8 • **Week 12**	Change in body weight	HbA_1c_ ^**m**^, random glucose
Carrizo 2009 [43], Venezuela	Test whether extended release metformin improves the metabolic profile of patients receiving CLZ	Patients of a schizophrenia outpatient centre (n = 61)	• Receiving CLZ for > 3 months preceding		• Age 18 and over	• Duration: 14 weeks • Metformin • Dose: 500mg/day for 2 weeks increased to 1000mg/day for 12 weeks	Placebo	• Week 7 • **Week 14**	**Change in body weight**	**HbA**_**1c**_, fasting glucose
Chen 2012 [44], US ^n^ **(excluded from meta-analysis)**	Test the effects of switching from current APM to aripiprazole or ziprasidone for patients with SMI and evidence of insulin resistance	Outpatients of three community-based clinical centres (n = 52)	• DSM-IV diagnosis of schizophrenia, schizoaffective disorder or bipolar disorder • Receiving APM treatment (no previous ARIP / ZIP)		• Age 18 to 64 • Evidence of insulin resistance	• Duration: 52 weeks • Aripiprazole • Dose: 5 to 30mg/day titrated to reach target dose within 2 weeks	Ziprasidone, dose: 40 to 160mg/day titrated to target dose in 2 weeks	• Week 6 • Week 12 • Week 26 • **Week 52**	Change in body weight	HbA_1c_
Chen 2013 [45], Taiwan	Test the effectiveness of metformin for controlling metabolic abnormalities in CLZ-treated patients with schizophrenia	In- and out-patients from psychiatric rehabilitation wards and two outpatient clinics (n = 55)	• DSM-IV diagnosis of schizophrenia or schizoaffective disorder • Receiving CLZ for > 3 months preceding	Excluded diabetes diagnoses or diabetic fasting plasma glucose levels	• Age 20 to 65 • BMI ≥ 24 • Fulfilled at least 1 criteria of MetS • Excluded weight loss medications	• Duration: 24 weeks • Metformin • Dose: 500mg twice daily (1000mg/day) in week 1 increased to 500mg 3 times daily (1500mg/day) in week 2	Placebo	• Week 2 • Week 4 • Week 8 • Week 16 • **Week 24** • 24 weeks after end	**Change in body weight**	Fasting plasma glucose
Deberdt 2005 [46], US **(excluded from meta-analysis)**	Examine efficacy of amantadine for managing OLZ induced weight gain in patients with SMI	In- and outpatients (n = 125)	• DSM-IV diagnosis of schizophrenia, schizoaffective, schizophreniform, or bipolar I disorders • Receiving OLZ for 1–24 months		• Age 18 to 65 • Gained ≥ 5% of body weight during first 9 months of OLZ therapy	• Duration: 16 weeks • Amantadine • Dose: 100mg/day for 2 weeks then increased in 100mg/day increments to 300mg/day if deemed appropriate	Placebo	• Week 12 • **Week 16**	**Change in body weight**	Fasting glucose
Deberdt 2008 [47], US	Examine the effects of switching from OLZ to quetiapine in obese patients with schizophrenia	26 centres (n = 133)	• DSM-IV diagnosis of schizophrenia or schizoaffective disorder • Receiving fixed dose of OLZ (10–20mg/day)		• Age 18 to 75 • BMI ≥ 30 • **or** BMI ≥ 25 and ≥ 1 CVD risk factor (including diabetes)	• Duration: 24 weeks • Quetiapine • Dose: 300–800mg/day	Care as usual: OLZ (7.5–20mg/day)	• Week 24	Time to relapse	HbA_1c_ fasting glucose
Fadai2014 [48] ^**o**^, Iran	Assess whether saffron aqueous extract (SAE) or crocin prevents OLZ induced MetS and insulin resistance in patients with schizophrenia	Inpatient ward of one psychiatric hospital (n = 66), 3 arm trial	• DSM-IV diagnosis of schizophrenia • No history of OLZ treatment		• Male • Age 18 to 65 • Excluded patients with MetS	• Duration: 12 weeks • OLZ 5–20mg/day PLUS • **Arm 1: SAE** • Dose: 15mg twice daily • **Arm 2: Crocin** • Dose: 15mg twice daily	OLZ 5–20mg/day PLUS **Placebo (Arm 3)**	• Week 2 • Week 6 • **Week 12**	**Presence of MetS** ^p^	HbA_1c_, **fasting glucose** ^p^
Fan 2013 [49], US	Examine effects of aripiprazole on metabolic parameters in CLZ treated patients with schizophrenia	Outpatients from a mental health centre (n = 38)	• DSM-IV diagnosis of schizophrenia or schizoaffective disorder • Treatment with CLZ for > 1 year and stable dose		• Age 18 to 65	• Duration: 8 weeks • Aripiprazole • Dose: 15mg/day	Placebo	• Week 8	**Glucose metabol-ism** FSIVGTT	HbA_1c_, fasting plasma glucose
Fleischhacker 2010 [50], 10 European countries and South Africa	Examine effects of adjunctive aripiprazole on body weight and metabolic parameters in CLZ treated patients with schizophrenia	Outpatients from 55 centres across countries involved (n = 207)	• DSM-IV diagnosis of schizophrenia • Receiving stable dose of CLZ monotherapy (200–900mg/day) ≥ 3 months (not optimally controlled)		• Age 18 to 65 • Experienced weight gain of ≥ 2.5kg while taking CLZ	• Duration: 16 weeks • Aripiprazole • Dose: 5mg/day for 2 weeks with an option to increase to 10mg/day in weeks 2–4, and to 15mg/day after week 4	Placebo	• Weeks 1–4 • Week 6 • Week 8 • Week 12 • **Week 16**	**Change in body weight**	Fasting glucose
Graham 2005 [51], US **(excluded from met-analysis)**	Examine effects of amantadine on weight gain and metabolic profile in psychiatric patients taking OLZ	Outpatients from private clinics and schizophrenia treatment programme (n = 21)	• A psychiatric condition (not specified) • Receiving OLZ treatment		• Gained ≥ 5lbs while taking OLZ	• Duration: 12 weeks • Amantadine • Dose: up to 300mg/day	Placebo	• Week 12	**BMI**	Fasting glucose
Henderson 2005 [52], US	Examine effectiveness of sibutramine to attenuate OLZ induced weight gain in patients with schizophrenia	Outpatients from an adult clinic of an urban mental health centre (n = 37)	• DSM-IV diagnosis of schizophrenia or schizoaffective disorder • Taking stable dose of OLZ ≥ 4 months		• Age 18 to 65 • BMI ≥ 30 • **or** BMI ≥ 27 and ≥ 1 CVD risk factor (including diabetes) • Excluded weight loss medications	• Duration: 12 weeks • Sibutramine • Dose: Weeks 1 to 4–5mg twice daily (could be reduced to once daily in response to side effects), weeks 5 to 12—increased to 15mg/day as tolerated	Placebo	• Week 4 • Week 8 • **Week 12**	**Weight loss**	**HbA**_**1c**_, random glucose
Henderson 2007 [53], US	Investigate efficacy of sibutramine for weight loss in CLZ treated patients with schizophrenia	Outpatients from a CLZ clinic of an urban mental health centre (n = 21)	• DSM-IV diagnosis of schizophrenia or schizoaffective disorder • Taking stable dose of CLZ ≥ 4 months		• Age 18 to 65 • BMI ≥ 30 • **or** BMI ≥ 27 and ≥ 1 CVD risk factor • Excluded weight loss medications	• Duration: 12 weeks • Sibutramine • Dose: Weeks 1 to 4–5mg twice daily • Weeks 5 to 12—increased to 15mg/day as tolerated (adjusted to 1–3 capsules daily)	Placebo	• Week 12	Weight loss	HbA_1c_, fasting glucose
Henderson 2009A [54] ^**q**^, US	Investigate effects of adjunctive aripiprazole to attenuate OLZ induced weight gain in patients with schizophrenia	Outpatients from an urban community mental health clinic (n = 15^s^)	• DSM-IV diagnosis of schizophrenia or schizoaffective disorder • Maintained on stable dose of OLZ for ≥ 1 month		• Age 18 to 65 • BMI ≥ 30 • **or** BMI ≥ 27 and ≥ 1 CVD risk factor (including diabetes)	• Duration: 10 weeks • Week 1 to 4: Aripiprazole • Dose: 15mg/day • Week 5 to 6: Washout • Week 7–10: Placebo	Week 1 to 4: Placebo, Week 5 to 6: Washout, Week 7–10: Aripiprazole (15mg/day)	**Week 4** • Week 6 • Week 10	**Change in body weight**	HbA_1c_, fasting glucose
Henderson 2009B [55], US	Investigate effects of rosiglitazone for CLZ induced glucose metabolism impairment in patients with schizophrenia	Outpatients of a mental health clinic (n = 18)	• DSM-IV diagnosis of schizophrenia or schizoaffective disorder • Treated with CLZ for ≥ 1 year	Excluded patients with diabetes	• Age 18 to 65 • Evidence of insulin resistance or impaired glucose (≥ 110mg/dl) • Excluded weight loss medications	• Duration: 8 weeksv • Rosiglitazone • Dose: 4mg/day	Placebo	• Week 8	Insulin resistance FSIVGTT	HbA_1c_, fasting glucose
Hoffmann 2012 [56] ^**r**^, Israel, Mexico, Republic of Korea, Russian Federation, and US	Determine if OLZ induced weight gain can be managed with adjunctive treatment algorithms that include amantadine, metformin and zonisamide	Outpatients (n = 199), 3 arm trial	• DSM-IV diagnosis of schizophrenia or schizoaffective disorder • Patients who were treatment resistant to OLZ were excluded	Excluded patients with diabetes	• Age 18 to 65 • BMI between 20 and 35 inclusive	• Duration: 22 weeks • OLZ 5–20mg/day PLUS: • **Arm 1:** amantadine (200mg/day) with option of switching to metformin (500–1500mg/day) and then zonisamide (100–400mg/day) • **Arm 2:** metformin with option of switching to amantadine & zonisamide (same doses as Arm 1)	**Arm3:** OLZ 5–20mg/day	• Week 22	Change in body weight	**HbA**_**1c**_ ^**s**^, fasting glucose
Holka-Pokorska [57], Poland	Evaluate the effects of a steroid hormone (DHEA) on MetS parameters in patients with schizophrenia treated with OLZ	Setting not reported (n = 55)	• ICD-10 diagnosis of schizophrenia or schizoaffective disorder • No prior treatment with CLZ • Receiving stable dose of OLZ ≥ 6 weeks		• Male • Age 18 to 65	• Duration: 12 weeks • Dehydro-epiandrosterone • Dose: 50mg/day for first 2 weeks, then titrated up to 100mg/day for remaining weeks if tolerated	Placebo	• Week 12	MetS criteria	**Fasting glucose**
Jarskog 2013 [58], US	Determine whether metformin promotes weight loss in overweight patients with schizophrenia	Outpatients from 17 academic, VA, and private clinic mental health sites (n = 148)	• DSM-IV diagnosis of schizophrenia or schizoaffective disorder • Duration of illness ≥ 1 year • Receiving stable dose (> 2 months) of no more than 2 APMs	Excluded patients with diabetes	• Age 18 to 65 • BMI ≥ 27 • Excluded previous treatment with metformin • Excluded weight loss medications in past month	• Duration: 16 weeks • Metformin • Dose: 500mg twice daily increased to 1500mg/day after week 1 and to 2000mg/day at week 3	Placebo	• Week 16	**Change in body weight**	**HbA**_**1c**_, fasting glucose
Joffe 2008 [59], Finland	Investigate effects of adjunctive orlistat to attenuate OLZ or CLZ induced weight gain in patients with SMI	Inpatients or outpatients (n = 71)	• SMI diagnosis that was relatively stable • Receiving stable dose of CLZ or OLZ	Excluded patients with type 1 diabetes	• Age 18 to 65vBMI between 28 and 43 inclusive • Excluded patients with significant changes in body weight in last 4 weeks	• Duration: 16 weeks • Orlistat • Dose: 120mg capsule taken during the main fat-containing meals	Placebo	• Week 4 • Week 8 • **Week 16**	Change in body weight	Fasting glucose
Karagianis 2009 [60] ^**t**^, Canada, Netherlands, US and Mexico	Investigate effects of orally disintegrating OLZ to attenuate standard OLZ induced weight gain in patients with SMI	Outpatients (n = 149)	• DSM-IV diagnosis of schizophrenia, schizoaffective, schizophreniform or bipolar disorders, or other related psychotic disorder • Taking OLZ (standard tablets) (5–20mg) for between 4 and 52 weeks		Experienced weight gain of ≥ 5kg or a change of ≥ 1 kg/m^2^ BMI • Excluded patients using weight loss medication / programme	• Duration: 16 weeks • OLZ orally disintegrating form (5–20mg) PLUS Placebo (tablet) • Dose: as per previous OLZ standard treatment (5–20mg)	Continue with OLZ standard tablets PLUS Placebo (sublingual)	• Week 16	Change in BMI	HbA_1c_, fasting glucose
Kusumi 2012 [61], Japan	Investigate effects of orally disintegrating OLZ on body weight and metabolic measures in OLZ-naïve patients with schizophrenia	Patients of 7 general, 12 psychiatric and 1 university hospital (n = 118)	• DSM-IV diagnosis of schizophrenia • Required a change in APM or new treatment with APM			• Duration: 12 months • Drug: OLZ orally disintegrating form (5–20mg) • Dose: Flexible dose prescribed by treating psychiatrists	OLZ standard tablets, dose: Flexible dose prescribed by treating psychiatrist	• Month 3 • Month 6 • Month 12	Change in body weight	HbA_1c_, fasting glucose
Lee 2013 [62], Taiwan	Investigate effects of adjunctive memantine on metabolic parameters in patients with bipolar II disorder using valproate	Outpatient and inpatient settings (n = 135)	• DSM-IV diagnosis of bipolar II disorder			• Duration: 12 weeks • Valproate (500 or 1000mg/day)vPLUS • Memantine • Dose: 5mg/day	Valproate (500 or 1000mg/ day) PLUS Placebo	• Week 2 • Week 8 • **Week 12**	Psychotic symptom severity	HbA_1c_, fasting serum glucose
Li 2013 [63], US	Examine effect of adjunctive insulin therapy on metabolic function in patients with schizophrenia treated with APM	Outpatients from an urban community mental health clinic (n = 45)	• DSM-IV diagnosis of schizophrenia or schizoaffective disorder • Receiving stable dose of APM for ≥ 1 month	Excluded patients with diabetes	• Age 18 to 65	•Duration: 8 weeks • Intranasal insulin treatment (40IU) • Dose: 4 times daily	Placebo	• Week 8	Change in body weight	HbA_1c_, fasting plasma glucose
Lu 2004 [64], Taiwan	Investigate effects of adjunctive fluvoxamine to attenuate CLZ induced weight gain and metabolic abnormalities in patients with schizophrenia	Inpatients (n = 68)	• DSM-IV diagnosis of schizophrenia • Treatment resistant to typical APMs • Not receiving CLZ or other atypical APM		• Age 18 to 60	• Duration: 12 weeks • CLZ and fluvoxamine co-administration • Dose: CLZ ≤ 250mg/day (low dose) individually titrated; fluvoxamine 50mg/day	CLZ monotherapy, dose: ≤ 600mg/day individually titrated	• Week 12 • Body weight measured weekly	Change in body weight	**Fasting serum glucose**
McElroy 2012 [65], US	Examine effects of zonisamide for attenuating OLZ induced weight gain in patients with SMI	Outpatients of one mental health centre (n = 42)	• DSM-IV diagnosis of a psychotic or bipolar disorder for whom OLZ would be clinically indicated • No treatment with OLZ within last 3 months		• Age ≥ 18 • BMI ≥ 22 • Excluded patients using weight loss medication	• Duration: 16 weeks • OLZ (5–25mg/day adjusted for optimal response) PLUS Zonisamide • Dose: 100mg/day titirated to maximum 600mg/day over 6 weeks	OLZ (5–25mg/day adjusted for optimal response) PLUS Placebo	• Week 16	**Change in body weight**	Fasting glucose
Modabbernia 2014 [66], Iran	Assess efficacy of melatonin for attenuating the metabolic side-effects of OLZ in patients with first-episode schizophrenia	Patients of one academic psychiatric hospital (n = 48)	• DSM-IV diagnosis of schizophrenia and in their first-episode of illness • Eligible for OLZ treatment and no prior OLZ in last 3 months	Excluded patients with diabetes	• Age 18 to 65 • Excluded patients with MetS	• Duration: 8 weeks • OLZ (5mg/day titrated to 25mg/day for optimal response) PLUS Melatonin • Dose: 3mg/day	OLZ (5mg/day titrated to 25mg/day for optimal response) PLUS Placebo	• Week 4 • **Week 8**	**Change in body weight**	Fasting glucose
Narula 2010 [67], India	Assess efficacy of topiramate for preventing weight gain and metabolic side-effects of OLZ in patients with first-episode schizophrenia	Inpatients and outpatients attending the psychiatry clinic at a tertiary care hospital (n = 72)	• ICD-10 diagnosis of schizophrenia • In their first-episode of illness and drug-naïve		• Age 18 to 65	• Duration: 12 weeks • OLZ (5mg-20mg/day titrated for optimal response) PLUS Topiramate • Dose: 50mg/day for 1 week and then 100mg/day	OLZ (5mg- 20mg/day titrated for optimal response) PLUS Placebo	• Week 12	**Change in body weight**	**Fasting glucose**
Newcomer 2008 [68], Multi-national	Examine effects of switching from OLZ to aripiprazole on metabolic parameters in overweight patients with schizophrenia	Multi-centre (n = 173)	• DSM-IV diagnosis of schizophrenia or schizoaffective disorder • Receiving OLZ monotherapy (10–20mg/day) at screening, for between 1 and 24 months	Excluded patients with diabetes	• Age 18 to 65 • BMI ≥ 27 • Weight gain during OLZ treatment verified • Excluded patients who lost > 10% of body weight in last 3 months	• Duration: 16 weeks • Aripiprazole • Dose: Titrated to 15mg/day over 2 weeks with down-titration of OLZ treatment; after 4 weeks flexible dosed at 10–30mg/day for optimal response	Care as usual: OLZ (same dose for 4 weeks then dosed at 10–20mg/day for optimal response)	• Week 4 • Week 8 • Week 12 • **Week 16**	**Change in body weight**	Fasting plasma glucose
Smith2013 [69] ^**u**^, US and China	Examine effects of pioglitazone on metabolic abnormalities in patients with schizophrenia treated with APM	4 sites in US and 1 site in China (n = 56)	• DSM-IV diagnosis of schizophrenia or schizoaffective disorder (chronic) • Currently treated with any APM	Impaired fasting glucose (≥ 100mg/dl) or current treatment with anti-diabetic medication	• Impaired lipids (triglycerides ≥ 120mg/dl and/or HDL <40mg/dL)	• Duration: 3 months • Pioglitazone • Dose: 30mg/day with permission to raise to 45mg/day if glucose control was deemed insufficient	Placebo	• Month 1 • Month 2 • **Month 3**	**Fasting glucose**^**v**^	HbA_1c_, **fasting glucose**^**v**^
Stroup 2011 [70], US	Examine efficacy of switching from OLZ, quetiapine or risperidone to aripiprazole for ameliorating metabolic risk in patients with schizophrenia	27 clinical research centres affiliated with the Schizophrenia Trials Network (US) (n = 215)	• DSM-IV diagnosis of schizophrenia or schizoaffective disorder • Stable dose of OLZ (5–20mg), quetiapine (200–1200mg) or risperidone (1–16mg) ≥ 3 months		• BMI ≥ 27 • Impaired lipids (non-HDL cholesterol ≥ 130mg/dl) • Desire to improve metabolic risk profile	• Duration: 24 weeks • Aripiprazole • Dose: 5mg/day in week 1; 10mg/day in week 2; 10–15mg/day in week 3; optimised to 5–30mg/day from week 4 • Previous APM was removed over 4 weeks	Care as usual: OLZ, quetiapine or risperidone continued at previous dose	• Week 4 • Week 8 • Week 12 • Week 16 • Week 20 • **Week 24**	**Non-HDL-C**	HbA_1c_, fasting glucose
Tek 2014 [71], US	Examine effects of adjunctive naltrexone to counteract APM-associated weight gain in patients with schizophrenia	Setting not reported (n = 24)	• DSM-IV diagnosis of schizophrenia or schizoaffective disorder • Receiving stable dose of APM		• Female • Age 18 to 70 • BMI ≥ 27 and >2% of body weight gain in last year • Excluded weight loss medications	• Duration: 8 weeks • Naltrexone • Dose: 25mg/day	Placebo	• Week 8	**Change in body weight**	HbA_1c_, random glucose
Wang 2012 [72], China	Evaluate efficacy of metformin for treatment of APM induced weight gain in patients with first-episode schizophrenia	Outpatients of a schizophrenia clinic in two hospitals (n = 72)	• DSM-IV diagnosis of schizophrenia and in first-episode of illnessvReceiving stable dose of only one APM • Have been treated with OLZ, sulpiride, CLZ, or risperidone	Excluded patients with diabetes	• Age 18 to 60 • Gained >7% of body weight in first year of being treated with OLZ, CLZ, risperidone or sulpiride	• Duration: 12 weeks • Metformin • Dose: 250mg twice daily for 3 days increased to 500mg twice daily (1000mg/day) for remaining period	Placebo	• Week 4 • Week 8 • **Week 12**	**Change in body weight**	Fasting glucose
Wani 2015 [73], India	Examine efficacy of switching from OLZ to aripiprazole to improve metabolic profile of patients with schizophrenia and MetS	Outpatients of a tertiary care psychiatry hospital (n = 62)	• DSM-IV diagnosis of schizophrenia • Achieved clinical stability with OLZ • Receiving OLZ (10–20mg/day) for ≥ 3 months with no other APM for ≥ 1 month		• Fulfilling criteria for presence of MetS • Desire to improve their metabolic risk profile	• Duration: 24 weeks • Aripiprazole • Dose: 5mg/day in week 1; 10mg/day in week 2; 10–15mg/day in week 3; 10–20mg/day in week 4 and 10–30mg/day from week 5 • OLZ removed over 4 weeks	Care as usual: OLZ (previous dose)	• Week 8 • **Week 24**	**Presence of MetS**	Fasting plasma glucose
Wu 2008B [74], China	Assess efficacy of metformin to prevent OLZ induced weight gain in drug-naïve patients with schizophrenia	New inpatients (first episode) at one hospital (n = 40)	• DSM-IV diagnosis of schizophrenia and in first-episode of illness • No APM usage for at least 3 months	Excluded patients with diabetes	• Age 18 to 50	• Duration: 12 weeks • OLZ (15mg/day) PLUS Metformin • Dose: 250mg three times daily (750mg/day)	OLZ (15mg/day) PLUS Placebo	• Week 4 • Week 8 • **Week 12**	**Change in body weight**	Fasting plasma glucose

Table footnotes:

Abbreviations: APM = antipsychotic medication; ARIP = aripiprazole; BMI = body mass index (kg/m^2^); CLZ = clozapine; CV = cardiovascular; CVD = cardiovascular disease; DSM-IV = Diagnostic and Statistical Manual of Mental Disorders, 4^th^ Edition; FSIVGTT = frequently sampled intravenous glucose tolerance test; HbA_1c_ = glycated haemoglobin; HDL = high-density lipoprotein; ICD-10 = International Statistical Classification of Diseases and Related Health Problems, 10^th^ Revision; kg = kilograms; m = metre; MetS = metabolic syndrome; MH = mental health; MHP = mental health professional; non-HDL-C = total cholesterol minus high-density lipoprotein; NR = not reported; OGTT = oral glucose tolerance test; OLZ = olanzapine; OT = Occupational Therapist; PPA = phenylpropanolamine; SAE = saffron aqueous extract; SMI = severe / serious mental illness; ZIP = ziprasidone

^a^ Follow-up highlighted in bold is end of intervention follow-up and is the time point used for the meta-analyses

^b^ Outcomes highlighted in bold indicate statistically significant improvement for intervention compared to control

^c^ Attux 2013: results for change in body weight only significant at 6 months

^d^ Cordes 2014: results for body weight only significant for % change; results for fasting glucose and OGTT only significant at 48 weeks

^e^ Daumit 2013: no p-value or indication of statistical significance provided

^f^ Forsberg 2008: cluster design including staff and patient participants–patient data used for meta-analysis

^g^ Gillhoff 2010: results for BMI only significant in women

^h^ Mauri 2008: intervention participants receive intervention for full 24 weeks, control participants receive intervention in weeks 13 to 24 –data collected at week 12 used for meta-analysis

^i^ Poulin 2007: no data or results reported for HbA_1c_

^j^ Weber 2006: no data or results reported for fasting glucose because of problems obtaining data

^k^ Wu 2008: data were used in both meta-analyses–for pharmacological interventions we combined the metformin arms and compared these to the placebo arms; for behavioural interventions we compared the lifestyle plus placebo arm to the placebo arm

^l^ Amrami-Weizman 2013: data pooled from two trials, however trials were identical in design and conduct

^m^ Borovicka 2002: no data or results reported for HbA_1c_

^n^ Chen 2012: this study included two similar interventions and no control group and was therefore excluded from the meta-analysis

^o^ Fadai 2014: the two intervention arms were pooled for the meta-analysis due to the same mechanism of action

^p^ Fadai 2014: both arms were superior to placebo

^q^ Henderson 2009A: cross-over design–data from week 4 used for meta-analysis (before cross-over)

^r^ Hoffmann 2012: the two intervention arms were pooled for the meta-analysis due to the same mechanism of action

^s^ Hoffmann 2012: only arm B was superior to the control intervention

^t^ Karagianis 2009: standard deviation for mean change scores were imputed from Kusumi 2012 because of data error

^u^ Smith 2013: the US and China sites were separated for the meta-analysis due to conflicting results (paper reports US results only)

^v^ Smith 2013: significance refers to findings from US sites

### Participants

There were a total of 4,392 participants; 2,315 were assigned to intervention and 2,077 to control arms.

Participants were mainly drawn from mental health outpatient and inpatient settings (n = 47 studies). One study recruited from supported housing schemes [24], and one from residential care facilities and day programmes [29]. Five studies did not report setting [47, 57, 68, 69, 71].

All but three studies [48, 57, 71] included both men and women, although overall women were under-represented (41%). Mean age ranged from 25 to 53 years, with a mean age across studies of 43 years. Ethnicity was poorly recorded, but varied significantly due to the range of countries included.

Eighteen studies recruited participants with schizophrenia; 20 with schizophrenia and schizoaffective disorder; two with bipolar disorder; and 14 with various SMIs. The majority of studies included clinically stable participants who had been diagnosed for several years.

Inclusion of participants with diabetes varied. Only one study specifically recruited people with type 2 diabetes [29]. Twenty-three studies excluded participants with diabetes (one excluded type 1 diabetes [59]). The remaining studies did not specify this in eligibility criteria. Mean HbA_1c_ at baseline ranged from 4.1% to 7.4% (n = 26 studies); 13 studies reported a mean in the American Diabetes Association (ADA) pre-diabetes category (5.7–6.4%) and two in the diabetes range (≥6.5%). Mean fasting glucose ranged from 4.3 to 6.8mmol/L (n = 43 studies); 14 studies reported a baseline mean in the ADA high risk category (5.6–6.9mmol/L) [75].

Thirty studies targeted overweight participants or those who had experienced significant weight gain. Mean BMI at baseline ranged from 20.2 to 41.9Kg/m^2^ (n = 49 studies); 41 studies reported a mean BMI of over 25Kg/m^2^.

### Interventions

Of the 54 RCTs identified, 40 assessed a pharmacological, 13 a non-pharmacological and one a mixed intervention.

Among the pharmacological studies, 32 used a placebo in the control arm [35–43, 45, 46, 48–55, 57–60, 62, 63, 65–67, 69, 71, 72, 74]. Seven trials evaluated an intervention against usual care [47, 56, 61, 64, 68, 70, 73]. One study compared two interventions but did not include a control arm [44].

Among the non-pharmacological studies, six compared an intervention with usual care [21, 22, 25, 27, 30, 32], and three provided basic information or advice to controls at baseline [23, 26, 29]. In one study, the intervention was also given to the control group after week 12 of 24 weeks [28]. Two studies included an active control arm [24, 31]. One trial did not describe the control intervention [33].

The mixed intervention study included four arms: metformin, metformin plus a lifestyle intervention, lifestyle plus placebo, and a control arm receiving placebo alone [34].

#### Pharmacological interventions

In total, 23 different medications from 15 categories of drug were evaluated (see Table 2).

**Table 2:** Pharmacological Intervention Categories.

Category and drugs	Mechanism of action
**Diabetes medication n = 12 [34, 36, 37, 39, 43, 45, 55, 58, 63, 69, 72, 74]**	
Insulin	Diabetes treatment used to regulate carbohydrate and fat metabolism in the body.
Metformin	A biguanide used to prevent and treat Type 2 diabetes by increasing insulin sensitivity and reducing the amount of glucose produced and released by the liver.
Pioglitazone, Rosiglitazone	Thiazolidinediones help to regulate glucose and fat metabolism by improving insulin sensitivity, allowing insulin to work more effectively. Rosiglitazone has been withdrawn from the EU.
**Weight loss medication n = 9 [35, 40, 46, 51–53, 59, 65, 67]**	
Amantadine	A dopamine agonist approved to treat extrapyramidal side effects and parkinsonian, with potential to decrease prolactin (plays a role in metabolism) or to decrease appetite.
Orlistat	An anti-obesity drug which acts on the gastro-intestinal tract by reducing absorption of dietary fat.
Reboxetine	An anti-depressant, believed to promote weight loss by inhibiting serotonin re-uptake and by doing so regulate eating behaviour and appetite control.
Sibutramine	A centrally acting appetite suppressant which has been withdrawn from the UK and other countries.
Topiramate, Zonisamide	Anticonvulsant (epileptic) medication believed to promote weight loss by stimulating energy expenditure and decreasing body fat stores (by inhibiting carbonic anhydrase).
**Weight loss and diabetes combination n = 2 [38, 56]**	
Amantadine + metformin + zonisamide, Metformin + amantadine + zonisamide	Treatment algorithms of anti-diabetic, anti-parkinsonian and anti-epileptic medications to allow patients to switch between treatments depending on clinical response.
Metformin + sibutramine	Adding an appetite suppressant to an anti-diabetic may enhance weight loss potential.
**Antipsychotic switching n = 10 [44, 47, 49, 50, 54, 60, 61, 68, 70, 73]**	
Aripiprazole, Quetiapine, Ziprasidone	Switching to or adding an atypical antipsychotic associated with fewer metabolic side effects is hypothesised to alleviate weight gain and metabolic abnormalities caused by the more commonly used antipsychotics like olanzapine and clozapine.
Olanzapine orally disintegrating	The orally disintegrating form of olanzapine is argued to induce fewer metabolic side effects than the standard tablet.
**Other n = 8 [41, 42, 48, 57, 62, 64, 66, 71]**	
Crocin, Saffron aqueous extract (SAE)	Herbal extracts with the potential to enhance lipid profile and metabolic function. Crocin is the active ingredient of SAE.
Dehydroepiandrosterone (DHEA)	A steroid hormone with systemic anti-atherosclerotic properties which help to increase insulin sensitivity and prevent development of metabolic syndrome components.
Fluvoxamine	An anti-depressant used in combination with clozapine could help to reduce clozapine dose thereby alleviating APM induced weight gain and metabolic side effects.
Melatonin, Ramelteon	Hypnotics used to treat insomnia which act on the circadian rhythm (normal sleep-wake cycle) and are believed to be important metabolic regulators.
Memantine	Used to treat dementia, memantine has an anti-depressant like and mood stabilizing effect and is believed to reduce binge eating episodes and weight.
Naltrexone	An opioid receptor antagonist believed to promote weight loss by altering the food reward system disturbed by APM treatment, in particular decreasing craving for sweet foods.
Phenylpropanolamine (PPA)	A stimulant used as a decongestant and anorectic agent which has been withdrawn from the UK and other countries. Its anorexiant actions are thought promote weight loss.

In nine studies, all participants in intervention and control arms were also enrolled onto a lifestyle programme [38, 39, 44, 51–53, 58, 69, 70]. In seven further studies all participants had lifestyle advice at baseline [43, 45, 46, 56], personal wellness counselling [65], limited dietary intake [74], or mandatory monthly dietary counselling [42]. Details of these interventions and levels of engagement were not reported.

Intervention duration varied from four weeks to 12 months, being 6 months or less in most studies.

#### Non-pharmacological interventions

All 14 non-pharmacological interventions targeted change in individual behaviour rather than organisation of care. Interventions were variously described as lifestyle interventions, weight loss programmes and physical exercise programmes; however, there was considerable overlap between these categories. In total, eight interventions included regular exercise sessions [23–25, 27, 30, 31, 33, 34] and three restricted calorie intake [28, 33, 34]. All but one intervention [31] included dietary recommendations, and all but two [31, 33] employed educational and behavioural strategies promoting a healthier lifestyle.

Staff delivering interventions varied, but the majority were mental health staff. No intervention specifically included carers of participants, although in one, carers were invited to join a session [21].

Intervention duration varied from 12 weeks to 18 months, with the majority being between 4 and 6 months. Group sessions were provided in 12 interventions, 4 of which also included individual sessions or follow-up calls. Sessions varied from 30 minutes to 2 hours in length, with frequency ranging from 3-times weekly to once a month.

### Outcomes

Our primary outcomes of interest were HbA_1c_ and fasting glucose.

Nineteen pharmacological studies measured both of these outcomes. A further five measured HbA_1c_ (one did not provide data) [42], and 16 measured fasting glucose (one did not provide data [51] and one provided dichotomous data that were not useable in meta-analysis [46]).

Three behavioural studies measured both HbA_1c_ and fasting glucose [24, 29, 30]. One of these did not provide data for HbA_1c_ [30], and one reported log transformed data for fasting glucose which were not useable in meta-analysis [29]. One study measured HbA_1c_ only [25], and the other nine studies measured fasting glucose only (one did not provide data [32] and one provided dichotomous data that were not useable [26]).

The mixed intervention study only measured fasting glucose.

All studies measured HbA_1c_ and/ or fasting glucose at the end of the intervention period. Details of the primary outcomes and follow-up period for each study are shown in Table 1.

### Risk of bias

The risk of bias assessment for each study is provided in S2 Table. Only one study was assessed as low risk across all domains [74]. Reporting of trial design was limited in many studies. Attrition was a particular problem for behavioural interventions and also for antipsychotic switching trials, many of which reported higher discontinuation rates in the intervention compared to control groups.

### Effectiveness of interventions

For HbA_1c_, six of 28 (five pharmacological and one behavioural), and for fasting glucose, nine of 48 studies (five pharmacological, three behavioural and one mixed intervention) showed improvement in the intervention group compared to the control interventions. The remainder reported no difference between groups (see Table 1).

### Meta-analysis

In the 48 trials included, there were a total of 4,052 participants; 2,150 were assigned to intervention and 1,902 to control arms.

#### Pharmacological interventions

**Fig 2:**
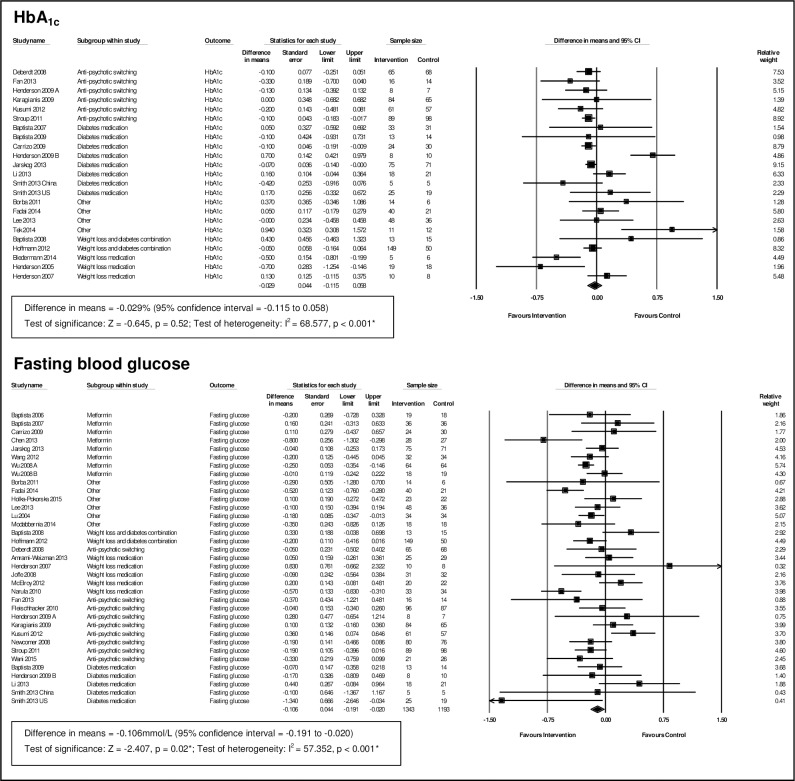
Meta-analysis of pharmacological interventions.

For pharmacological interventions, we pooled data from 22 studies for HbA_1c_ (n = 1515) and 34 for fasting glucose (n = 2536) (see Fig 2).

For HbA_1c_ there was no evidence of a difference between the intervention and control groups (MD = -0.03%; 95% Confidence Interval (CI) [-0.12, 0.06]; p = 0.52). Results, however, were heterogeneous (I^2^ = 69%).

For fasting glucose there was a small but statistically significant improvement of -0.11mmol/L (95% CI, [-0.19, -0.02]; p = 0.02) for the intervention group compared to controls. Again, there was heterogeneity (I^2^ = 57%). Investigation of baseline imbalance (see S1 Fig) showed that the control group had slightly lower levels of fasting glucose (MD = 0.07mmol/L; 95% CI, [0.01, 0.14]; p = 0.03), a difference that while statistically significant, was very small, and if anything would lead to underestimation of the overall effect size.

For subgroup analysis of pharmacological interventions, we used the drug type categories described earlier (see Table 2). For the ‘diabetes medication’ category, we further subdivided interventions into ‘metformin’ and ‘other diabetes’ treatment. Meta-analysis (see Table 3) showed that antipsychotic switching (MD = -0.11%; 95% CI, [-0.18, -0.05]; p = 0.001; I^2^ = 0%) and metformin (MD = -0.08; 95% CI, [-0.14, -0.03]; p = 0.004; I^2^ = 0%) were effective in lowering HbA_1c_ compared to placebo or usual care, albeit with modest effect sizes. For fasting glucose, only metformin was effective (MD = -0.15mmol/L; 95% CI, [-0.29, -0.01]; p = 0.04; I^2^ = 51%).

**Table 3:** Results of Subgroup Analyses.

Subgroup analysis	Number of studies	Meta-analysis					Heterogeneity		
		Difference in means	95% confidence intervals (CI)		Standard error	p-value^a^	I^2^ (%)	p-value	
**PHARMACOLOGICAL INTERVENTION SUBGROUP ANALYSES**									
**Intervention categories–HbA**_**1c**_ **(%)**									
Metformin	3	-0.08	-0.14	-0.03	0.03	**0.004**	0	0.81	
Other diabetes medications	5	0.15	-0.22	0.53	0.19	0.42	78	0.001	
Weight loss medications	3	-0.32	-0.84	0.20	0.27	0.22	86	0.001	
Antipsychotic switching	6	-0.11	-0.18	-0.05	0.03	**0.001**	0	0.86	
Weight loss and diabetes combinations	2	-0.02	-0.24	0.20	0.11	0.84	8	0.30	
**Intervention categories–fasting blood glucose (mmol/L)**									
Metformin	8	-0.15	-0.29	-0.01	0.07	**0.04**	51	0.04	
Other diabetes medications	5	-0.06	-0.44	0.33	0.20	0.78	45	0.12	
Weight loss medications	5	-0.06	-0.44	0.32	0.19	0.77	79	0.001	
Antipsychotic switching	9	-0.04	-0.20	0.12	0.08	0.62	45	0.07	
Weight loss and diabetes combinations	2	0.04	-0.47	0.56	0.26	0.87	83	0.02	
**Exclusion of people with diabetes–HbA1c (%)**									
***All pharmacological interventions*:**									
excluding people with diabetes	9	0.14	-0.04	0.32	0.09	0.13	76		<0.001
not excluding people with diabetes	14	-0.11	-0.21	-0.01	0.05	**0.04**	59		0.003
***Diabetes medication interventions (including metformin)*:**									
excluding people with diabetes	5	0.18	-0.14	0.50	0.17	0.27	87		<0.001
not excluding people with diabetes	3	-0.11	-0.31	0.09	0.10	0.29	26		0.26
**Exclusion of people with diabetes–fasting blood glucose (mmol/L)**									
***All pharmacological interventions*:**									
excluding people with diabetes	19	-0.08	-0.18	0.01	0.05	0.09	52		0.004
not excluding people with diabetes	16	-0.14	-0.31	0.03	0.09	0.12	64		<0.001
***Diabetes medication interventions (including metformin)*:**									
excluding people with diabetes	10	-0.12	-0.26	0.02	0.07	0.08	53		0.02
not excluding people with diabetes	3	-0.30	-1.12	0.52	0.42	0.48	50		0.13
***Weight loss medication interventions*:**									
excluding people with diabetes	2	0.13	-0.08	0.34	0.11	0.21	0		0.48
not excluding people with diabetes	3	-0.23	-0.77	0.30	0.27	0.39	66		0.05
***Antipsychotic switching interventions*:**									
excluding people with diabetes	2	-0.04	-0.33	0.24	0.15	0.78	56		0.13
not excluding people with diabetes	7	-0.04	-0.25	0.17	0.11	0.71	51		0.06
**BEHAVIOURAL INTERVENTION SUBGROUP ANALYSES**									
**Intervention duration–fasting blood glucose (mmol/L)**									
Short interventions (6 months or less)	6	-0.23	-0.34	-0.12	0.06	**<0.001**	0		0.56
Long interventions (longer than 6 months)	4	-0.50	-0.74	-0.25	0.13	**<0.001**	31		<0.001
**Repeated physical activity–fasting blood glucose (mmol/L)**									
Interventions with repeated physical activity	7	-0.33	-0.52	-0.14	0.10	**0.001**	55		0.04
Interventions with no physical activity	3	-0.11	-0.36	0.14	0.13	0.40	0		0.85
**Exclusion of people with diabetes–fasting blood glucose (mmol/L)**									
Studies excluding people with diabetes	3	-0.28	-0.40	-0.15	0.06	**<0.001**	0		0.70
Studies not excluding people with diabetes	7	-0.28	-0.53	-0.03	0.13	**0.03**	61		0.02

Table footnotes:

^**a**^ p-values highlighted in bold indicate statistically significant effects at the 0.05 level.

Subgroup analyses of studies that excluded participants with diabetes at baseline, and those that did not, showed that pharmacological interventions were effective in lowering HbA_1c_ only in the mixed population (MD = -0.11%; 95% CI, [-0.21, -0.01]; p = 0.04; I^2^ = 59%). For fasting glucose, neither group showed a statistically significant improvement compared to controls (Table 3). The meta-regression found no association between baseline HbA_1c_ or fasting glucose levels and effect size (see S3 Fig).

To explore this further, we repeated the subgroup analysis for certain categories of pharmacological interventions: diabetes medication, weight loss medication and antipsychotic switching for fasting glucose; and diabetes medication for HbA_1c_. No group showed statistically significant improvements compared to controls (Table 3). We observed larger effect sizes in studies that did not exclude diabetes at baseline for the diabetes medication and weight loss medication categories, but similar effects for antipsychotic switching (Table 3). We did not have sufficient data to examine the remaining categories.

#### Behavioural interventions

**Fig 3:**
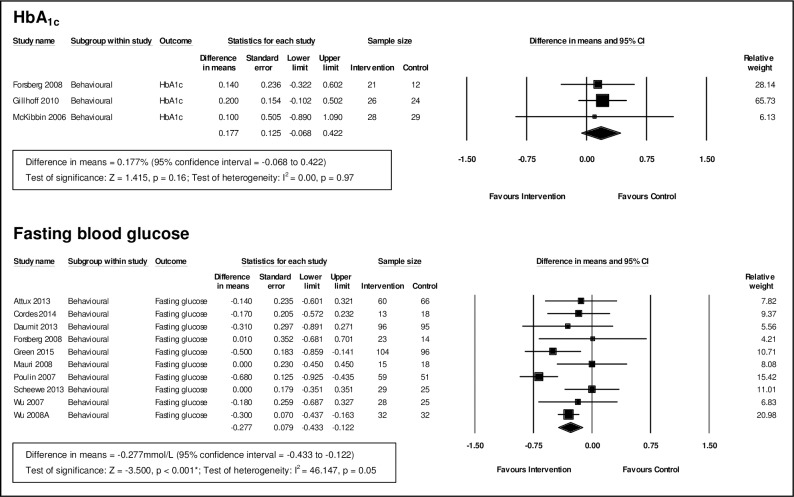
Meta-analysis of behavioural interventions.

For behavioural interventions, we pooled data from three studies for HbA_1c_ (n = 140) and 10 for fasting glucose (n = 956) (see Fig 3).

Behavioural interventions were not found to be effective in lowering HbA_1c_, (MD = 0.18%; 95% CI, [-0.07, 0.42]; p = 0.16).

For fasting glucose, there was evidence of a difference of -0.28mmol/L (95% CI, [-0.43, -0.12]; p<0.001) comparing behavioural interventions with controls. Although there was evidence of heterogeneity (I^2^ = 46%), 7 of the 10 studies favoured the intervention. Investigation of baseline imbalance (see S2 Fig) showed that controls had slightly lower levels of fasting glucose (MD = 0.10mmol/L; 95% CI, [-0.02, 0.23]; p = 0.10).

For subgroup analysis and meta-regression of behavioural interventions, we only examined fasting glucose due to the small number of studies measuring HbA_1c_. Participants receiving an intervention that included physical activity showed an improvement in fasting glucose of -0.33mmol/L (95% CI, [-0.52, -0.14]; p = 0.001; I^2^ = 55%) compared to usual care (see Table 3). Participants receiving an intervention for 6 months or less had lower fasting glucose compared to usual care (MD = 0.23mmol/L; 95% CI, [-0.34, -0.12]; p<0.001; I^2^ = 0%); interventions of more than 6 months duration showed an even greater effect, lowering fasting glucose by 0.50mmol/L (95% CI, [-0.74, -0.25]; p<0.001; I^2^ = 31%), (Table 3). The meta-regression (see S4 Fig) confirmed that interventions of a longer duration had a greater effect on fasting glucose compared to usual care (coefficient = -0.006; 95% CI, [-0.01, -0.002]; p = 0.007).

Subgroup analysis of studies that excluded (MD = -0.28mmol/L; 95% CI, [-0.40, -0.15]; p<0.001; I^2^ = 0%) or did not exclude people with diabetes at baseline (MD = -0.28mmol/L; 95% CI, [-0.53, -0.03]; p = 0.03; I^2^ = 61%) showed similar statistically significant effects of behavioural interventions in improving fasting glucose compared to controls (Table 3). However, the meta-regression (S4 Fig) showed that effect size increased with higher baseline fasting glucose, suggesting that interventions may be more effective in those with poorer glycaemic control (coefficient = -0.36; 95% CI, [-0.59, -0.13]; p = 0.002).

### Sensitivity analyses

**Fig 4:**
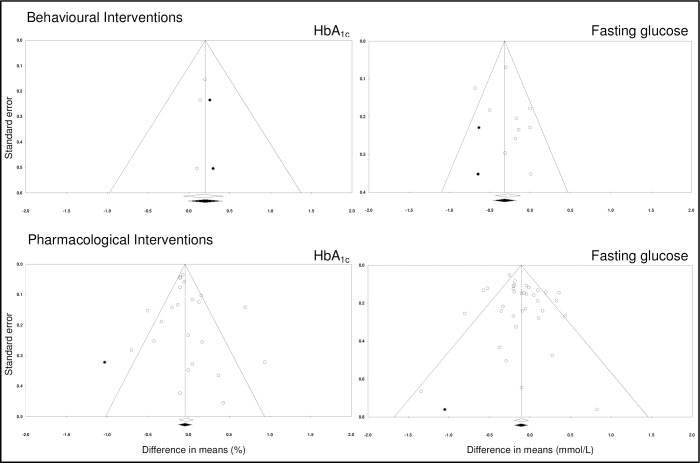
Funnel plots of behavioural and pharmacological studies.

Leave-one-out analyses showed that no single study had a disproportionate effect on each of the main meta-analyses. However, funnel plots showed some asymmetry (see Fig 4), suggesting potential publication bias for both the behavioural and pharmacological literature.

The trim-and-fill analysis suggests there is some evidence of missing studies (shown as black on the funnel plots in Fig 4).The adjusted effect sizes, accounting for publication bias are presented in Table 4. Publication bias adjusted effect sizes suggest that pharmacological interventions reduce both HbA_1c_ and fasting glucose, and behavioural interventions are effective in reducing fasting glucose but not HbA_1c_.

**Table 4:** Results of Trim-and-fill Analysis.

Analysis	Number of studies	Meta-analysis			Trim-and-fill effect size (95% CI) [adjusted studies]	I^2^
		Difference in means	95% confidence intervals (CI)			
**Behavioural**						
*HbA*_*1c*_ *(%)*	3	0.18	-0.07	0.42	0.20 (-0.01 to 0.41) [5]	0%
*Fasting glucose (mmol/L)*	10	-0.28^b^	-0.43	-0.12	-0.32 (-0.46 to -0.17) [12]	46%
**Pharmacological**						
*HbA*_*1c*_ *(%)*	23^a^	-0.03	-0.12	0.06	-0.04 (-0.13 to -0.05) [24]	69%
*Fasting glucose (mmol/L)*	35^a^	-0.11^b^	-0.19	-0.02	-0.11 (-0.19 to -0.02) [35]	57%

Table footnotes:

^**a**^ The China and US sites in Smith (2013) are counted as separate studies.

^**b**^ Significant at the 0.05 level.

## Discussion

### Summary of evidence

Overall, compared to usual care, both pharmacological and behavioural interventions improved fasting glucose levels, but not HbA_1c_ in people with SMI, with behavioural interventions showing a larger difference compared with pharmacological interventions. However, after adjusting for publication bias, there was some evidence that pharmacological interventions may also improve HbA_1c_. Subgroup analyses showed improvements in HbA_1c_ for antipsychotic switching and metformin; and in fasting glucose for metformin. For behavioural interventions, those that included regular physical activity were more effective in lowering fasting glucose than those that did not. Subgroup analysis and meta-regression showed that interventions of longer duration resulted in greater improvements in fasting glucose compared to usual care, and this may help to explain why the small number of studies measuring HbA_1c_ did not show an improvement, as only one of these was greater than 6 months in duration.

Some categories of pharmacological interventions (diabetes and weight loss medications), appeared to have a smaller effect on lowering glycaemic measurements in studies that excluded people with diabetes at baseline compared to the effect observed in studies that did not. However, it was not possible to investigate this robustly because of limited data, and the meta-regression of all pharmacological interventions showed no association between baseline levels of HbA_1c_ or fasting glucose and effect size. For behavioural interventions, studies that included participants with higher baseline glucose levels appeared to be more effective in a meta-regression, although the subgroup analysis showed no difference between studies that excluded those with diabetes compared to those that did not.

Our findings are consistent with previous meta-analyses. Bruins *et al*., [7] found a significant improvement in fasting glucose levels with lifestyle interventions (standardised MD = -0.24, 95% CI, [-0.32, -0.10]; p = 0.001; n = 8 studies; I^2^ = 0%) but did not include HbA_1c_ in their analysis or explore intervention characteristics. Mizuno *et al*., [6] explored pharmacological strategies to counteract the metabolic side effects of antipsychotic medication, and reported statistically significant improvements in fasting glucose for metformin (MD = -0.18mmol/L; 95% CI, [-0.35, 0.00]; n = 9 studies; I^2^ = 73%); and in HbA_1c_ for metformin (MD = -0.08%; 95% CI, [-0.13, -0.03]; n = 3 studies; I^2^ = 0%) and aripiprazole (MD = -0.65%; 95% CI, [-1.25, -0.06]; n = 2 studies; I^2^ = 89%). Our findings were similar but included drugs in addition to aripiprazole in the antipsychotic switching group.

In common with these previous reviews, we found the improvements reported in HbA_1c_ and fasting glucose were modest. However, there was considerable heterogeneity in results. Differences in effect sizes and direction of effect between studies made it difficult to assess the overall effectiveness of interventions. Several studies showed a reduction in fasting glucose and at the same time an increase in HbA_1c_ or vice versa [37–39, 43, 54]. These results are difficult to explain because logically one would expect a corresponding change, particularly in longer duration studies which would take account of the time required to alter HbA_1c_. However, there are a number of trials that demonstrate that although HbA_1c_ and fasting glucose are well correlated, they do not always respond in similar ways [76].

For people with SMI, this relationship may be complicated further by the metabolic side effects of anti-psychotic medication, which will work against interventions designed to improve glycaemic control [77]. For example, in several of the pharmacological and behavioural intervention studies, fasting glucose or HbA_1c_ increased in both the intervention and control groups, but with a smaller increase in the intervention group [24, 31, 43, 49, 56]. Through subgroup analysis and meta-regression, we have been able to identify certain intervention and population characteristics that may explain some of the differences in effect between studies, and identify particular interventions that show the most promise. However, these findings should be viewed within the context of methodologically limited trials, and for the antipsychotic switching and behavioural interventions, substantial dropout in follow-up.

### Limitations

Although we included a larger number of studies compared with previous reviews, a limitation of our findings relates to the quantity and quality of evidence included, and the substantial risk of potential bias identified in included studies. We were also unable to fully explore differential effects between those with and without diabetes, or to compare our findings to evidence in the general population because of the lack of data to measure onset of diabetes in those without diabetes, and diabetic complications in those with diabetes. Previous reviews have also commented on the paucity and poor quality of evidence in this area [10, 14]. Strengths of our review include a published protocol, robust search, independent screening and data extraction by at least two reviewers, and the use of appropriate meta-analytic methods to explore the results.

### Implications for clinical practice

These results suggest that antipsychotic switching strategies, metformin, sustained behavioural interventions, and behavioural interventions that include regular physical activity offer the greatest potential to improve glycaemic control in the SMI population. Whilst the effect sizes were modest, such improvements in glycaemic control can help to avoid onset of diabetes and attenuate diabetes complications [11], therefore, the small differences reported in key subgroups may still be clinically significant. Also, combining pharmacological and behavioural strategies may incrementally (or perhaps even synergistically) increase effectiveness [34]. However, the effect sizes observed were modest when compared to the general population [18, 78], suggesting that tailored interventions which address the specific challenges faced by people with SMI are needed.

In real world settings, the SMI population will face challenges in adhering to new medications or engaging in sustained behavioural interventions involving attendance at regular group sessions [23, 79]. These challenges will likely be compounded when implementing multifaceted interventions. Moreover, we need to reflect carefully before pursuing adjunctive pharmacological therapies in a population for whom polypharmacy is already problematic; and the potential acceptability of switching from an antipsychotic medication providing clinical stability to one which may help to improve physical health, but for which the efficacy in preventing relapse in mental illness is uncertain. These considerations, along with the sparse evidence base, mean that recommendations for clinical practice remain limited. Nonetheless, this review does provide some evidence to support current practice of providing lifestyle interventions and switching to antipsychotics with a better metabolic profile in people with SMI.

## Conclusions

Improving diabetes outcomes in SMI is a global priority, but the evidence-base to guide clinical practice is limited. Despite the challenges described above, a number of pharmacological and behavioural approaches warrant further exploration. Metformin is already a well-established treatment in diabetes [17]. Its use alongside antipsychotic prescriptions to prevent diabetes merits further investigation. Switching of antipsychotic medication is also common in clinical practice. Research is needed to understand which antipsychotics offer the greatest potential benefit, and to optimise dosage and timing of such interventions in order to reduce glycaemic burden, whilst maintaining clinical stability for people with SMI.

Behavioural interventions show perhaps more promise than pharmacological strategies, but little is known about the behaviour change techniques that might be most effective for people with SMI and diabetes. This is a key area for research, if we are to avoid ever-increasing inequalities in health and access to healthcare, as diabetes management becomes increasingly predicated on self-management. Future research should focus on the design of appropriate interventions, and test the potential acceptability and feasibility of delivering them in a real world setting, before establishing effectiveness in a trial evaluation.

## Supporting Information

S1 PRISMA Checklist (DOC)

S1 Appendix Example of search strategy. (DOCX)

S1 Table Summary of on-going studies. (DOCX)

S2 Table Risk of bias assessment for included studies. (DOCX)

S1 Fig Meta-analysis of baseline imbalance in pharmacological studies. (DOCX)

S2 Fig Meta-analysis of baseline imbalance in behavioural studies. (DOCX)

S3 Fig Meta-regression of the difference in means for pharmacological interventions by A) baseline HbA_1c_ and B) baseline fasting glucose.
(DOCX)

S4 Fig Meta-regression of the difference in mean fasting glucose for behavioural interventions by (A) intervention duration (B) baseline fasting glucose. 7
(DOCX)
