# Supplementary material for: The Effectiveness of Pharmacological and Non-Pharmacological Interventions for Improving Glycaemic Control in Adults with Severe Mental Illness: A Systematic Review and Meta-Analysis
Source: PLoS One. 2017 Jan 5;12(1):e0168549. doi: 10.1371/journal.pone.0168549 (PMC5215855; doi:10.1371/journal.pone.0168549)
Supplement: S1 Appendix — (DOCX) [file pone.0168549.s002.docx]

**S1 Appendix – Example of search strategy**

Search strategies from published Cochrane reviews related to diabetes, schizophrenia and depression were used to inform the search concepts [[1-3](#_ENREF_1)]. We also used the Cochrane Highly Sensitive Search Strategy for identifying randomized trials in Medline: “sensitivity- and precision maximising version” (2008 revision) [[4](#_ENREF_4)].

The search strategy for Ovid MEDLINE is provided below:

**Ovid MEDLINE(R) <1946 to November Week 3 2014>**

11-12-14

1 diabet*.tw. (409057)

2 exp Diabetes mellitus/ (331256)

3 (non insulin* depend* or noninsulin* depend* or non insulin?depend* or noninsulin?depend*).tw. (11975)

4 (insulin* depend* or non insulin?depend*).tw. (28057)

5 (IDDM or NIDDM or MODY or T1DM or T2DM or T1D or T2D).tw. (25850)

6 or/1-5 (465042)

7 exp Diabetes Insipidus/ (7173)

8 diabet* insipidus.tw. (6806)

9 7 or 8 (8978)

10 6 not 9 [diabetes] (457783)

11 Mental Disorders/ (125673)

12 chronic disease/ or recurrence/ (386322)

13 11 and 12 (4661)

14 (bipolar adj (disorder* or disease* or illness*)).tw. (17359)

15 depressi* symptom*.tw. (30722)

16 Depression/ (82520)

17 major depression.tw. (18085)

18 exp schizophrenia/ (88929)

19 capgras syndrome/ (432)

20 mood disorders/ or affective disorders, psychotic/ or bipolar disorder/ or depressive disorder/ or depression, postpartum/ or depressive disorder, major/ or depressive disorder, treatment-resistant/ (120679)

21 delusional parasitosis/ (8)

22 morgellons disease/ (28)

23 paranoid disorders/ (3796)

24 exp psychotic disorders/ (39920)

25 exp Dyskinesia, Drug-Induced/ (6443)

26 exp Akathisia, Drug-Induced/ (1322)

27 exp Neuroleptic Malignant Syndrome/ (1864)

28 exp Movement Disorders/ (112090)

29 schizo*.tw. (104582)

30 hebephreni*.tw. (264)

31 oligophreni*.tw. (872)

32 (mani* adj3 depress*).tw. (6877)

33 "mental condition".tw. (374)

34 (psychotic* adj3 depress*).tw. (2102)

35 (severe* adj3 affective*).tw. (184)

36 (severe* adj3 mental*).tw. (7741)

37 (severe* adj3 depress*).tw. (7557)

38 (psychos#s adj3 depress*).tw. (2315)

39 (serious* adj3 affective*).tw. (41)

40 "serious mood*".tw. (21)

41 (serious* adj3 mental*).tw. (2997)

42 (serious* adj3 depress*).tw. (524)

43 (tardiv* adj dyskine*).tw. (3772)

44 (akathisi* or acathisi*).tw. (1585)

45 (neuroleptic adj5 malignant adj2 syndrome).tw. (1871)

46 (movement adj5 (disorder or disorders)).tw. (13185)

47 neuroleptic-induc*.tw. (1220)

48 parkinsoni*.tw. (21243)

49 exp Parkinson Disease/ (51177)

50 (parkinson?s adj disease).ti. (27782)

51 49 or 50 (52906)

52 48 not 51 (9824)

53 or/13-47,52 (466683)

54 randomized controlled trial.pt. (401171)

55 controlled clinical trial.pt. (90794)

56 randomized.ab. (296222)

57 placebo.ab. (155341)

58 clinical trials as topic.sh. (176780)

59 randomly.ab. (207517)

60 trial.ti. (129163)

61 54 or 55 or 56 or 57 or 58 or 59 or 60 (913392)

62 exp animals/ not humans.sh. (4099178)

63 61 not 62 (839457)

64 10 and 53 and 63 [RCTS] (718)

65 10 and 53 (7226)

66 limit 65 to "reviews (maximizes specificity)" [diabetes smi reviews] (258)

67 64 or 66 (922)

68 (exp child/ or exp infant/ or adolescent/) not exp adult/ (1573938)

69 67 not 68 [RCTS and Reviews] (882)

**References**

1. Baumeister H, Hutter N, Bengel J. Psychological and pharmacological interventions for depression in patients with diabetes mellitus and depression. Cochrane Database Syst Rev. 2012;12: Cd008381.

2. Pal K, Eastwood SV, Michie S, Farmer A, Barnard ML, Peacock R, et al. Computer-based interventions to improve self-management in adults with type 2 diabetes: a systematic review and meta-analysis. Diabetes Care. 2014;37(6): 1759-66.

3. Hunt GE, Siegfried N, Morley K, Sitharthan T, Cleary M. Psychosocial interventions for people with both severe mental illness and substance misuse. Cochrane Database Syst Rev. 2013;10: CD001088.

4. Lefebvre C, Manheimer E, Glanville J. Chapter 6: Searching for studies. 2011. In: Cochrane Handbook for Systematic Reviews of Interventions Version 5.1.0 [Internet]. [www.cochrane-handbook.org:](http://www.cochrane-handbook.org:) The Cochrane Collaboration.
