# Supplementary material for: The Effectiveness of Pharmacological and Non-Pharmacological Interventions for Improving Glycaemic Control in Adults with Severe Mental Illness: A Systematic Review and Meta-Analysis
Source: PLoS One. 2017 Jan 5;12(1):e0168549. doi: 10.1371/journal.pone.0168549 (PMC5215855; doi:10.1371/journal.pone.0168549)
Supplement: S1 Table — (DOCX) [file pone.0168549.s003.docx]

**S1 Table – Summary of on-going studies**

| **Name** | **Intervention type** | **Intervention aim** | **Intervention** | **Completion** | **Trial reference** |
| --- | --- | --- | --- | --- | --- |
| Chwastiak 2013 | Behavioural | To improve diabetes management in people with schizophrenia and Type 2 diabetes | Collaborative care | Jun-15 | ClinicalTrials.gov NCT02011529 |
| Gaughran 2013 | Behavioural | To improve metabolic outcomes and substance use in people with SMI | Health promotion (lifestyle) | Feb-13 | ISRCTN registry ISRCTN58667926 |
| Hahn 2013 | Behavioural | To improve diabetes management in people with SMI and Type 2 diabetes | Lifestyle intervention | Dec-15^a^ | ClinicalTrials.gov NCT01828931 |
| Holt 2015 | Behavioural | To promote weight loss and metabolic parameters in people with schizophrenia | Lifestyle intervention | Mar-17 | ISRCTN registry ISRCTN19447796 |
| Jerome 2012 | Behavioural | Increase cardiovascular fitness in adults with SMI | Physical activity with peer support | Dec-09 | ClinicalTrials.gov NCT00458094 |
| Jorg 2013 | Behavioural | To improve metabolic health in people with SMI residing in long-term care facilities | Lifestyle intervention | Nov-12 | http://www.trialregister.nl/ NTR2720 |
| Osborn 2013 | Behavioural | To reduce cardiovascular risk in patients with SMI | CVD risk management in primary care | Nov-15 | ISRCTN registry ISRCTN13762819 |
| Sajatovic 2011 | Behavioural | To improve diabetes management in people with schizophrenia and Type 2 diabetes | Diabetes self-management programme | Jul-15^a^ | ClinicalTrials.gov NCT01410357 |
| Voruganti 2005 | Mixed | To prevent diabetes in people with schizophrenia and pre-diabetes taking APM | Lifestyle intervention plus metformin | Jan-09 | ClinicalTrials.gov NCT00182494 |
| Barton 2012 | Pharmacological | To alleviate the metabolic side effects of APM in people with schizophrenia | Moxonidine | Dec-14 | ClinicalTrials.gov NCT01567124 |
| De Valk 2013 | Pharmacological | To improve glycaemic control in people with schizophrenia and Type 2 diabetes | Liraglutide (additional to metformin) | Aug-16 | http://www.trialregister.nl/ NTR4352 |
| Hahn 2014 | Pharmacological | To improve glycaemic control in people with schizophrenia and Type 2 diabetes | Metformin | Dec-16 | ClinicalTrials.gov NCT02167620 |
| Ishoy 2014 | Pharmacological | To alleviate APM induced weight gain in people with schizophrenia | Exenatide (GLP-1) | Jun-15^a^ | ClinicalTrials.gov NCT01794429 |
| Larsen 2014 | Pharmacological | To improve glycaemic control in people with SMI and pre-diabetes taking APM | Liraglutide (GLP-1) | Sep-15^a^ | ClinicalTrials.gov NCT01845259 |
| Meyer 2006 | Pharmacological | To alleviate APM induced weight gain in people with schizophrenia | Ziprasidone (APM switching strategy) | Dec-09^a^ | ClinicalTrials.gov NCT00338949 |
| Newcomer 2005 | Pharmacological | To alleviate the metabolic side effects of APM in people with schizophrenia | Aripiprazole (APM switching strategy) | Aug-09^a^ | ClinicalTrials.gov NCT00205660 |
| Rado 2007 | Pharmacological | To alleviate weight gain and metabolic side effects of APM in people with SMI | Metformin | Aug-11^a^ | ClinicalTrials.gov NCT00682448 |
| Tamminga 2008 | Pharmacological | To alleviate the metabolic side effects of APM in people with schizophrenia | Pramlintide | Aug-11^a^ | ClinicalTrials.gov NCT00690235 |
| Tek 2013 | Pharmacological | To alleviate weight gain and metabolic side effects of APM in people with SMI | Naltrexone | Feb-18 | ClinicalTrials.gov NCT01866098 |
| Abbreviations: APM = antipsychotic medication; CVD = cardiovascular disease; SMI = severe mental illness; ^a^ end of data collection period | | | | | |
