# Supplementary material for: The Effectiveness of Pharmacological and Non-Pharmacological Interventions for Improving Glycaemic Control in Adults with Severe Mental Illness: A Systematic Review and Meta-Analysis
Source: PLoS One. 2017 Jan 5;12(1):e0168549. doi: 10.1371/journal.pone.0168549 (PMC5215855; doi:10.1371/journal.pone.0168549)
Supplement: S2 Table — (DOCX) [file pone.0168549.s004.docx]

**S2 Table – Risk of bias assessment for included studies**
