# Supplementary material for: The Effectiveness of Pharmacological and Non-Pharmacological Interventions for Improving Glycaemic Control in Adults with Severe Mental Illness: A Systematic Review and Meta-Analysis
Source: PLoS One. 2017 Jan 5;12(1):e0168549. doi: 10.1371/journal.pone.0168549 (PMC5215855; doi:10.1371/journal.pone.0168549)
Supplement: S1 Fig — (DOCX) [file pone.0168549.s005.docx]

**S1 Figure – Meta-analysis of baseline imbalance in pharmacological studies**

**HbA_1c_**

**Fasting blood glucose**

Test of significance: Z = 0.417, p = 0.68

Test of heterogeneity: I^2^ = 57.125, p = 0.001*

Test of significance: Z = 2.150, p = 0.03*

Test of heterogeneity: I^2^ = 28.608, p = 0.06
