# Supplementary material for: The Effectiveness of Pharmacological and Non-Pharmacological Interventions for Improving Glycaemic Control in Adults with Severe Mental Illness: A Systematic Review and Meta-Analysis
Source: PLoS One. 2017 Jan 5;12(1):e0168549. doi: 10.1371/journal.pone.0168549 (PMC5215855; doi:10.1371/journal.pone.0168549)
Supplement: S2 Fig — (DOCX) [file pone.0168549.s006.docx]

**S2 Figure – Meta-analysis of baseline imbalance in behavioural studies**

**HbA_1c_**

**Fasting blood glucose**

Test of significance: Z = 1.187, p = 0.24

Test of heterogeneity: I^2^ = 0.000, p = 0.68

Test of significance: Z = 1.636, p = 0.10

Test of heterogeneity: I^2^ = 0.000, p = 0.90
