# Supplementary material for: The Effectiveness of Pharmacological and Non-Pharmacological Interventions for Improving Glycaemic Control in Adults with Severe Mental Illness: A Systematic Review and Meta-Analysis
Source: PLoS One. 2017 Jan 5;12(1):e0168549. doi: 10.1371/journal.pone.0168549 (PMC5215855; doi:10.1371/journal.pone.0168549)
Supplement: S3 Fig — Meta-regression of the difference in means for pharmacological interventions by A) baseline HbA1c and B) baseline fasting glucose. (DOCX) [file pone.0168549.s007.docx]

**S3 Figure – Meta-regression of the difference in means for pharmacological interventions by A) baseline HbA_1c_ and B) baseline fasting glucose**

1. **Baseline HbA_1c_**

Difference in mean HbA_1c_

Baseline HbA_1c_

Co-efficient = -0.211 (95% confidence interval = -0.496 to 0.075)

Test of significance: Z = -1.446, p = 0.148

1. **Baseline fasting glucose**

Difference in mean fasting glucose

Baseline fasting glucose

Co-efficient = -0.084 (95% confidence interval = -0.291 to 0.122)

Test of significance: Z = -0.802, p = 0.423
