# Supplementary material for: The Effectiveness of Pharmacological and Non-Pharmacological Interventions for Improving Glycaemic Control in Adults with Severe Mental Illness: A Systematic Review and Meta-Analysis
Source: PLoS One. 2017 Jan 5;12(1):e0168549. doi: 10.1371/journal.pone.0168549 (PMC5215855; doi:10.1371/journal.pone.0168549)
Supplement: S4 Fig — Meta-regression of the difference in mean fasting glucose for behavioural interventions by (A) intervention duration (B) baseline fasting glucose. 7 (DOCX) [file pone.0168549.s008.docx]

**S4 Figure – Meta-regression of the difference in mean fasting glucose for behavioural interventions by (A) intervention duration (B) baseline fasting glucose**

1. **Intervention duration**

Co-efficient = -0.006 (95% confidence interval = -0.01 to -0.002)

Test of significance: Z = -2.704, p = 0.007

Difference in mean fasting glucose

Intervention duration in weeks

1. **Baseline fasting glucose**

Co-efficient = -0.359 (95% confidence interval = -0.589 to -0.128)

Test of significance: Z = -3.047, p = 0.002

Difference in mean fasting glucose

Baseline fasting glucose
